# Cervical Myelopathy in Rheumatoid Arthritis

**DOI:** 10.1155/2011/153628

**Published:** 2011-11-22

**Authors:** N. Mukerji, N. V. Todd

**Affiliations:** Department of Neurosurgery, Regional Neurosciences Center, Royal Victoria Infirmary, Queen Victoria Road, Newcastle upon Tyne, NE1 4LP, UK

## Abstract

Involvement of the cervical spine is common in rheumatoid arthritis. Clinical presentation can be variable, and symptoms may be due to neck pain or compressive myeloradiculopathy. We discuss the pathology, grading systems, clinical presentation, indications for surgery and surgical management of cervical myelopathy related to rheumatoid arthritis in this paper. We describe our surgical technique and results. We recommend early consultation for surgical management when involvement of the cervical spine is suspected in rheumatoid arthritis. Even patients with advanced cervical myelopathy should be discussed for surgical treatment, since in our experience improvement in function after surgery is common.

## 1. Introduction

This paper will consider the surgical management of patients with rheumatoid arthritis (RA), in particular the management of RA patients with a cervical myelopathy.

RA is a chronic inflammatory disorder of joints. The aetiology is unknown. It is characterised by an erosive synovitis. Synovial inflammation can lead to joint erosions, erosions of periarticular soft tissues, and pannus formation. The cervical spine requires soft tissue integrity for stability. Damage to ligaments and joints can cause different types of instability [1, 2]. Radiological involvement of the cervical spine can be present in up to 86% of all RA patients [3, 4]. Instability can be associated with neck pain and/or it can be associated with compression of adjacent structures, particularly the brainstem, the spinal cord, or spinal nerve roots. Damage to the lateral masses of C1 leads to bone loss and vertical translocation of C1 through the foramen magnum, which is termed as basilar invagination. This can cause brainstem compression. The commonest instability is atlantoaxial (AA) subluxation, typically forward subluxation of C1 on C2 [5]. This can cause C2 root pain and/or a myelopathy. More than fifty percent of cervical spine deformity occurs at C1/2 [6, 7]. The remaining 50% occurs in the subaxial cervical spine and this can cause a radiculopathy or myelopathy. Subaxial instability at multiple levels produces a stepladder deformity. Instability is most commonly found in patients who have had RA for ten years or longer; many patients are asymptomatic over an extended period of time [8, 9].

Any orthopaedic surgery in patients with RA is challenging, given the potential problems of osteoporosis, immunocompromise and poor wound healing. If to those general problems we add the potentially severe neurological problems in RA patients with a myelopathy, we can see that RA myelopathic patients are a particular challenge.

## 2. Pathology

Synovitis develops in the facet joints, in the synovial tissue adjacent to the odontoid, and in uncovertebral joints at the lateral margins of the intervertebral discs. There is erosion of adjacent ligaments, the annulus, disc spaces, and bone [2, 10, 11].

There is progressive instability with typical radiographic features. As set out above, there are three patterns of instability which occur either singly, or together: the atlas shifts forward on the axis (atlantoaxial subluxation); one vertebral body shifts forward on the body of another at lower levels which may be seen at multiple levels and produces a stepladder deformity (subaxial subluxation); the axis telescopes into the atlas, driving the odontoid upwards (vertical subluxation or basilar invagination) [2]. Atlantoaxial (AA) subluxation results from erosive synovitis in the atlantoaxial, atlanto-odontoid, atlanto-occipital joints, the bursa between the odontoid and the transverse ligament. Superior migration of the odontoid is a consequence of erosion and bone loss in the occipitoatlantal and atlantoaxial joints. Subaxial subluxation results from destruction of the facets, intervertebral discs, and interspinous ligaments. Unlike degenerative disease, involvement of C2-C3 and C3-C4 is common, and osteophytes are seldom seen. AA subluxation, subaxial subluxation, and superior migration of the odontoid can be measured and graded [3, 12, 13].

## 3. Clinical Presentation

*Neck pain* may be a consequence of the primary inflammatory disorder. Neck pain is a potential consequence of subaxial instability. It can be difficult to distinguish these two causes of neck pain. We have relied upon the extent of peripheral joint disease. If an RA patient has a severe neck pain, if neck pain tends to occur at a time when peripheral joint disease is severe, and if it waxes and wanes with a similar pattern to the peripheral joint disease, we consider that, probably, neck pain is a consequence of the primary rheumatoid disorder. By contrast, where neck pain is severe but peripheral joint problems are mild then we consider that it is more likely that subaxial subluxation is the cause of neck pain. The localisation of neck pain in patients with RA is as difficult as it is in all patients with neck pain.

*Suboccipital pain*, typically a consequence of C2 nerve root involvement, is almost always associated with subluxation, most commonly, AA (C1-C2) subluxation, occasionally vertical subluxation. Subluxation, causing C2 radicular pain, is the commonest radiculopathy that occurs in RA [5, 14].

*Subaxial radiculopathy* does occur but it is relatively uncommon in RA patients.

*Myelopathy* is common. Patients present with the classical symptoms of gait disturbance, loss of fine motor control in the hands, numbness in the hands, and balance disturbance. It is held by some that it can be difficult to differentiate myelopathic problems in the hands from the problems that are a consequence of joint disease. Our experience has been different. We have found that most patients readily distinguish the long-term joint disorder from a new myelopathic disorder. Nevertheless we would agree that in some cases it may be difficult to distinguish between the two problems. In a similar fashion, neurological examination can be more difficult because of the rheumatoid joint involvement, particularly where there have been fusions, for example, of fingers or the wrist. We have found that careful neurological examination does allow us to attribute new functional problems to a new neurological disorder (as opposed to long-term persistent joint problems). The risk of an RA patient developing a myelopathy progressively increases as the residual canal diameter is reduced [15].

*Brainstem compression* is less common. It can produce facial sensory disturbance, dysphagia, or abnormalities in the lower cranial nerves. Objective signs of a myelopathy, including hyperreflexia, extensor plantar responses, positive Hoffman's signs or clonus, together with objective motor and sensory losses will be found in the majority of patients.

*Sudden death* is reported; it is rare [16].

*Deformity* can cause substantial disability in the absence of myelopathy, particularly where the deformity is severe, as in the chin-on-chest deformity.

## 4. Grading Systems: Clinical

In determining the severity of any disease process, the effects of surgical intervention, or the factors that influence prognosis and survival, it is helpful to have objective and reproducible means of measuring the patient's disability.

Pain can be reliably assessed by means of a visual analogue scale [17].

Various clinical grading systems have been used to describe the neurological (Ranawat classes I–IIIB, Table 1) [13] and functional (Steinbrocker's grades I–IV) [18, 19] status of RA patients. Other scales and scoring systems have been proposed [20].

## 5. Grading Systems: Radiological

Cervical instability can be assessed with flexion/extension plain X-rays and/or CT. It is crucial to establish whether instability is fixed or reducible. If there is fixed deformity decompression is usually needed prior to fixation/fusion. If the deformity is reducible, posterior fixation/fusion in extension reestablishes the normal canal diameter, successfully treating the patient.

The extent of AA subluxation can be assessed by the anterior or posterior atlantodental interval (AADI, PADI).

The *AADI* is the distance from the posterior margin of the anterior ring of C1 to the anterior surface of the odontoid. A distance of more than 3 mm in an adult or 4 mm in a child is abnormal [12, 21]. The AADI does not correlate well with the risk of developing a neurological deficit or the extent of any neurological deficit because patients have different primary canal diameters. The effect of a given degree of slip in a patient with a wide canal will be less than that in a patient whose canal is congenitally narrow.

The *PADI* is the distance from the posterior aspect of the odontoid to the anterior margin of the lamina of C1. The PADI is a good measure of the space available for the spinal cord in relation to the bony elements. The PADI is most accurately assessed with CT imaging in the subluxed, usually flexed, position. A PADI of 13 mm or less is associated with an increased risk of myelopathy [15, 22]. The space available for the cord may be less than the PADI as assessed on plain films or CT because soft tissue pannus may also contribute to cord compression.

*Subaxial subluxation* may be measured as either (i) the extent of subluxation measured in millimetres or (ii) the percentage of slip of one vertebra upon another. The sagittal diameter of the subaxial spinal canal better correlates with the presence and/or extent of myelopathy. Patients with subaxial canal diameters of 13 mm or less are at increased risk of myelopathy [15, 22].

**Figure 1:**
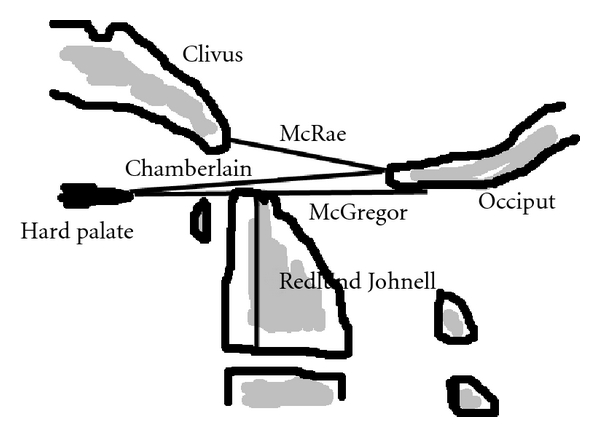
*Basilar invagination* is present when the odontoid tip is >6 mm above the Chamberlain's line; above McRae's line; >8 mm above McGregor's line in males and >9.7 mm above this line in females; or when the Redlund-Johnell distance is <34 mm in males and <29 mm in females.

There are several measurements that attempt to quantify the extent of vertical subluxation of the odontoid. No single measure has a high sensitivity or specificity; a combination of measurements has the greatest predictive power [23]. The four commonest measurements are those of McRae, McGregor, Chamberlain, and Redlund-Johnell [10] (Figure 1).

## 6. Surgical Considerations

The indications for surgical treatment include the following: (i) to treat C2 root pain; (ii) to prevent any myelopathy; (iii) to prevent further neurological deterioration in patients with a progressive cervical myelopathy; (iv) to treat deformity.

*Prophylactic surgery* to prevent any myelopathy is indicated where subluxation (typically A-A subluxation, but occasionally subaxial subluxation) has reduced the residual canal diameter to such an extent that there is a significant risk of a myelopathy developing in the future. It is the residual diameter of the spinal canal in the subluxed position that predicts myelopathy. The residual canal diameter is the bony PADI corrected for any additional soft tissue compromise. If the residual canal diameter is 13 mm or less, we would recommend prophylactic stabilisation [15, 22].

**Therapeutic Surgery**

Surgery can be indicated to treat deformity, and/or C2 root pain, and/or progressive myelopathy.

AA or subaxial deformity may be wholly asymptomatic. If the residual canal diameter is above 13 mm, such patients can be observed [15, 22]. Serial plain X-rays should be taken. If the deformity continues to progress, then that will often be an indication for stabilisation.

Deformity itself can be disabling, particularly where the deformity is severe as in the chin-on-chest deformity. Such a severe deformity can be painful; it can limit swallowing and eventually it can limit breathing. In such patients we would recommend reduction of the deformity in a halo, progressively, followed usually, by posterior long-segment stabilisation.

A-A subluxation is commonly associated with C2 radicular pain. In common with other cervical radiculopathies, pain can be severe and disabling. Posterior C1-C2 fixation and fusion lead to improvement or cure in C2 root pain in over 90% of cases [24, 25].

RA patients with a progressive cervical myelopathy typically require treatment to prevent progression of the myelopathy. Reduction of deformity and/or surgical decompression of the spinal cord usually prevents further neurological deterioration. There can be a surprising degree of functional recovery. Some have suggested that where an RA patient has a severe, nonambulant (Ranawat IIIB) myelopathy, surgical treatment is futile [24–27]. That is not our experience. We previously reported two cohorts of patients: (i) a group of eighteen nonambulant patients who, for a variety of reasons, were not referred for a surgical opinion and who were treated conservatively [28] and (ii) a consecutive group of thirty-two nonambulant patients who were referred for surgery and who were all operated upon regardless of the degree of neurological deficit [29]. The two groups cannot be compared directly because they were recruited at different points in time and in different ways. However, they were all Ranawat IIIB RA myelopathic patients. Of the eighteen patients who were treated conservatively, at six months 47% were dead, usually because of a complication of immobility such as bronchopneumonia or pulmonary embolism. The surgically treated patients did better. Of twenty-nine patients alive six months following surgery, twenty-four (83%) had improved neurologically (19 (65%), to Ranawat IIIA, 5 (17%) to Ranawat II) [29]. Our policy is to treat all myelopathic RA patients whatever the severity of the neurological deficit. Previously, Marks and Sharp [30] reported a 68% mortality in myelopathic RA patients treated conservatively. If one does not operate because one predicts a poor outcome, that prophecy will usually be fulfilled.

## 7. Preoperative Management

RA patients have comorbidities that are potentially a consequence of their disease and/or their age. Cardiac, pulmonary, and renal comorbidities should be assessed jointly by the surgical and anaesthetic staff. Any problems that can be corrected, should be. For transoral surgery the surgeon must assess the degree of mouth-opening (which may be reduced because of temporomandibular joint disease) and the presence/absence of permanent teeth. We have, once, had to abandon proposed transoral surgery because the transoral access proved to be insufficient.

We prefer (if time permits) to immobilise the patient in a halo preoperatively. This has some advantages: (i) safe intraoperative transfers and positioning; (ii) putting the patient into a fully functional position preoperatively; (iii) careful neurological observation during reduction of a deformity (in the halo). A halo put on three or four days preoperatively can be adjusted so that the best degree of vertical distraction and lordosis is attained in an ambulant and functioning patient. If, when reducing a deformity in a myelopathic patient there is an increased neurological deficit, the reduction can be reversed with, usually, recovery of neurological function. The authors are aware of several patients whose deformity has been reduced under anaesthetic immediately prior to decompression, fixation, and fusion where the patient has woken up quadriplegic. It is of course impossible to say which part of the procedure led to quadriplegia, but reduction of deformity intraoperatively is at least one potential cause, the risk of which can be minimised by reduction in a halo preoperatively. We have also found that putting the patient's head and neck in a functional position preoperatively is easier to judge than trying to do so intraoperatively with the patient prone.

## 8. Surgery

As for all spinal procedures, we need to consider (i) the approach; (ii) decompression; (iii) fixation; (iv) fusion. In RA patients the principles are not different from spinal patients as a whole although a special consideration is required in relation to fixation and fusion.

**Figure 2:**
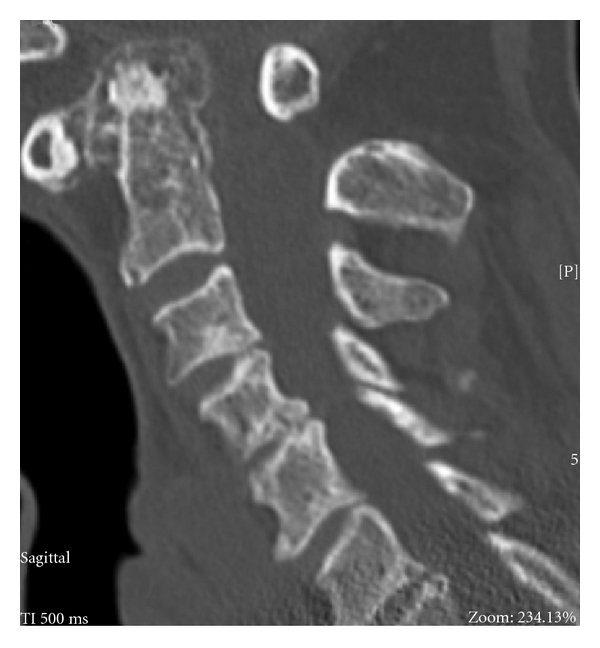
Sagittal CT scan reconstruction of a patient showing odontoid pannus reducing the diameter of the spinal canal at the level of the odontoid peg.

**Figure 3:**
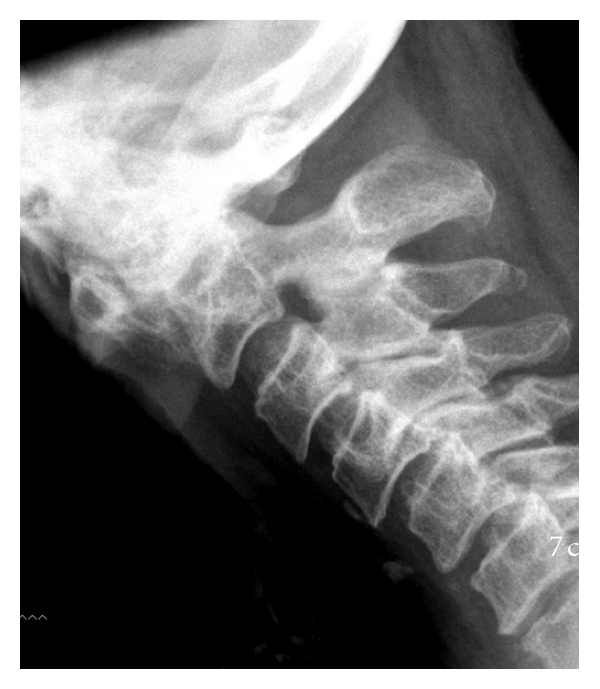
Lateral cervical radiograph of a patient demonstrating AA subluxation in flexion.

The *anterior* approach for *decompression* is appropriate for (i) anteriorly placed pathologies such as odontoid pannus (Figure 2) or subluxation of the odontoid (Figure 3); or subaxial disc/osteophyte disease; or kyphotic deformity.

A transoral odontoidectomy is a well-recognised and standardised procedure [31, 32]. It is usually safe. A key preoperative concern is to assess the extent of any rotatory deformity at C1/C2. Rotatory A-A deformity can bring the vertebral artery into the midline with injury to the vertebral artery if not recognised.

**Figure 4:**
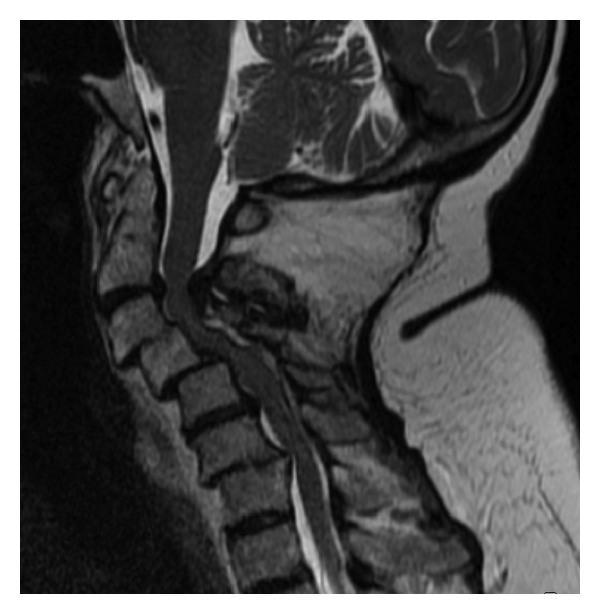
Sagittal MRI scan demonstrating the subaxial “staircase” deformity and significant posterior ligamentous hypertrophy contributing to cervical myelopathy.

In RA patients posterior excision of the arch of C1 is often required in patients with irreducible A-A subluxation. Subaxial laminectomies can be required where the myelopathy is, in part, from posterior ligamentous hypertrophy (Figure 4), but this is uncommon.

The number of levels to be decompressed and the approach are dictated solely by the clinical and radiological extent of spinal cord compression.

The nature, length, and position of *fixation* are crucially important. RA patients are, as a minimum, potentially unstable; most are actually unstable. Any actual or potential instability may be exacerbated by decompressive surgery. RA patients are commonly osteopaenic if not frankly osteoporotic. If the senior author (NVT) has made mistakes in his practice in relation to fixation in RA patients it is in underdoing, not overdoing the fixation. Fixation with screws into osteoporotic bone can lead to screw pullout; short segment posterior fixation, when long segment fixation is required; or posterior fixation only, when simultaneous anterior and posterior fixation were the preferred option which have all led to implant failure. A huge variety of implants are available; it is outwith the remit of this paper to review them all. Briefly, posterior fixation includes rods or occipital paddles fixed with screws, hooks, or wires. Screws can be into the occiput, directly into the lateral masses of C1 below and/or the pedicles of C2. Anterior fixation is typically via cages and/or anterior cervical plates and screws. Experienced spinal surgeons will have found methods that best suit themselves and their patients.

**Figure 5:**
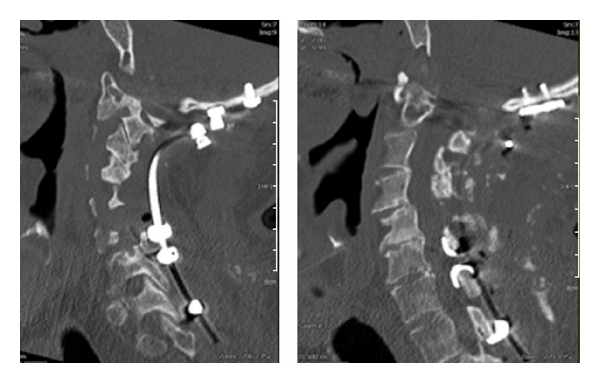
Occipitocervical fixation showing a double claw construct at C4/5 and C6/7 using sublaminar hooks.

**Figure 6:**
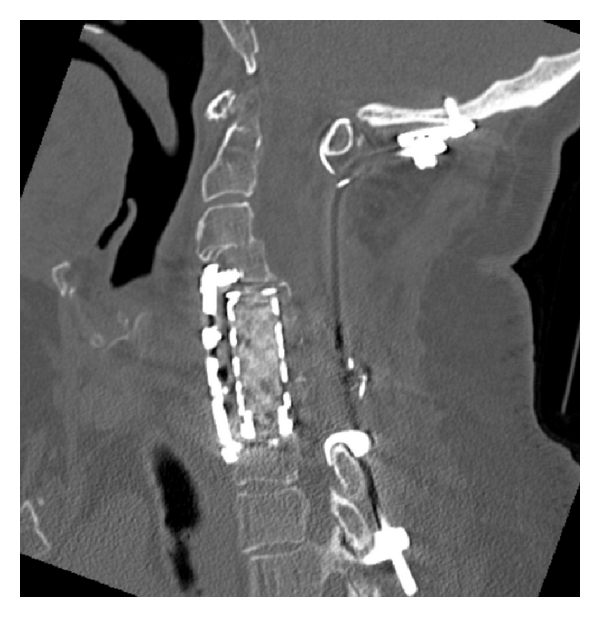
Occipitocervical fixation supplemented by vertebrectomy with an anterior cage and plate fixation for a patient who required decompressive cervical laminectomies.

Our most common construct is a posterior hook/rod construct (Figure 5). In the past we have experienced pullout when using screws in osteopaenic/osteoporotic bone. Osteoporosis is a disease of cancellous bone and the cervical laminae are largely cortical bones. Provided there is sufficient residual canal diameter to permit the use of sublaminar hooks we have found that hooks provide fixation that does not pull out. We prefer a double claw construct, typically at C4/5 and C6/7 (Figure 5). Where the laminae have been removed (which is unusual), a long segment posterior fixation will often need to be supplemented by anterior fixation/fusion (Figure 6). Where long segment posterior fixation has been used but fusion is only required over a short segment, the long segment fixation can be removed once the fusion is solid, permitting an increased range of movement.

For both reducible and irreducible A-A subluxation a variety of posterior constructs have been described. The key issue is to create a construct that controls the C1-C2 motion segment until solid fusion occurs.

For all fixations fusion is normally required. Internal fixation without fusion will usually fail in time, particularly in this group of patients, where bone quality is poor. As with other spinal patients, anterior fusions are more likely to be successful than posterior fusions, although posterior fixation and therefore fusion are more commonly needed in RA patients. For occiput-C1-C2 fixation/fusions onlay graft to the occiput and laminae of C1 and C2 is used. For posterior subaxial fusions, lateral mass fusions are standard. Autologous bone grafts are the gold standard, but long segment fusions (and indeed even some occiput-C1/C2 fusions) require more bones than is locally available (typically the available spinous processes). The surgeon then needs to decide whether to augment local bone with autologous bone, for example, from the iliac crest (which carries its own morbidity [33]) or whether to use bone expanders. We initially used entirely autologous bone but given donor site morbidity we now use local bone and expanders such as DBX (Demineralized bone matrix, Synthes Inc, Pa, USA) and/or Actifuse (Apatech Inc, Hertfordshire, UK) with good fusion rates in the main. If a posterior bone graft fails, it is better to repeat the fusion anteriorly, if that is possible; refusing into a previously failed posterior fusion bed has high failure rates.

## 9. Outcomes

Treatment of radiculopathy leads to high rates of improvement in radicular pain. In the commonest radiculopathy, the C2 radiculopathy from A-A subluxation, posterior stabilisation leads to improvement, and most commonly cure, of C2 root pain in over 90% of patients.

In patients with other compressive myelopathies, it is generally held that decompressive procedures halt neurological deterioration but improvement in neurological function is unpredictable [34, 35]. We might not expect better outcomes in RA but we have reported improvement in function in 83% of the worst affected RA patients (Ranawat IIIB, chair or bed-bound) [29] to the point that the improved patients could stand or walk. The six-month mortality was 9% in the surgically treated patients. We believe this mortality is acceptable when set against the high mortality of untreated Ranawat IIIB RA patients [27, 30]. We currently have a policy of treating all RA myelopathic patients surgically.

## 10. Conclusions

Cervical myelopathy in RA patients should be diagnosed and treated as early as possible to reduce preventable morbidity. However, diagnosis at an advanced stage should not preclude consideration of surgical treatment. Surgical considerations include (i) approach; (ii) decompression; (iii) fixation; (iv) fusion. We would recommend surgical treatment even in severe grades of myelopathy (Ranawat IIIB) to preserve life and improve function.

## Figures and Tables

**Table 1:** Ranawat grading of cervical myelopathy [13].

Class	Description
I	No neural deficit
II	Subjective weakness, dysaesthesia, and hyperreflexia
IIIA	Objective weakness and long-tract signs; patient remains ambulatory
IIIB	Objective weakness and long-tract signs; patient is no longer ambulatory
